# NOTCH1 Activation Depletes the Pool of Side Population Stem Cells in ATL

**DOI:** 10.13188/2377-9292.1000013

**Published:** 2017-06-14

**Authors:** Xue Tao Bai, Chien-Hung Yeh, Christophe Nicot

**Affiliations:** Department of Pathology and Laboratory Medicine, University of Kansas Medical Center, USA

**Keywords:** HTLV-I, ATL, Side population (SP) cells, NOTCH, PI3K

## Abstract

**Background:**

HTLV-I infection is associated with the development of adult T-cell leukemia (ATL), a malignancy characterized by a high rate of disease relapse and poor survival. Previous studies reported the existence of side population (SP) cells in HTLV-I Tax transgenic mouse models. These studies showed that these ATL-like derived SP cells have both self-renewal and leukemia renewal capacity and represent Cancer Stem Cells (CSC)/Leukemia-Initiating Cells (LIC). Since CSC/LIC are resistant to conventional therapies, a better characterization is needed.

**Methods:**

We isolated, sorted and characterized SP cells from uncultured PBMCs from ATL patients and from ATL patient-derived cell lines. We then identified several specific signaling pathways activated or suppressed in these cells. Expression of viral gene HBZ and Tax transcriptional activity was also investigated. Using gamma-secretase inhibitor (GSI, Calbiochem) and stably transduced ATL cell lines expressing TET-inducible NOTCH 1 intracellular domain (NICD), we characterized the role of activated NOTCH 1 in the maintenance of the SP cells in ATL.

**Results:**

Our studies confirm the existence of SP cells in ATL samples. These cells demonstrate lower activation of NOTCH1 and Tax, and reduced expression of STAT3, β-catenin/Wnt3 and viral HBZ. We further show that PI3K and the NOTCH1 signaling pathway have opposite functions, and constitutive activation of NOTCH1 signaling depletes the pool of SP cells in ATL-derived cell lines.

**Conclusions:**

Our results suggest that in ATL, a balance between activation of the NOTCH1 and PI3K signaling pathway is the key in the control of SP cells maintenance and may offer therapeutic opportunities.

## Introduction

Limiting dilution transplantation demonstrated that only a small percentage of cells within a cell line population can give rise to tumors *in vivo*. These observations suggest that tumors and cell lines are composed of cells that are heterogeneous in terms of tumor-forming potential [1,2]. Numerous studies demonstrate that side population (SP) cells identified by ABC pump-mediated exclusion of Hoechst can be referred to as Leukemia-initiating cells (LIC) or Cancer stem cells (CSC). These cells have the unique ability to regenerate full leukemia and self-renewal of the SP compartment in xenograft models [3]. SP analysis has also been used to identify CSC in a wide variety of human solid tumors, including breast, colon, ovarian and hepatic cancers [4–7]. These cells are relatively resistant to commonly used therapies. In addition, SP cells have also been reported in several hematologic malignancies, including but not limited to acute myeloid leukemia [8], chronic myeloid leukemia [9], and acute lymphoblastic leukemia (ALL) [10,11].

Human T-cell Leukemia Virus type I (HTLV-I) infection is associated with an aggressive and fatal form of T-cell leukemia/lymphoma known as adult T-cell leukemia/lymphoma (ATL) [12,13]. The mechanism by which HTLV-I engenders ATL is not fully elucidated, but numerous studies have demonstrated involvement of genetic and epigenetic events [14–18]. Overall, survival of ATL patients treated with various chemotherapy regimens is poor, with survival in several cohorts of patients presenting predominantly with acute leukemia or lymphoma ranging between 5.5 and 13 months [19]. Although most therapies initially result in a partial or complete remission, the vast majority of patients relapses and die, suggesting that current treatments do not completely eradicate ATL tumor cells. Consistent with these observations, published data suggest the existence of a slowly dividing cell subpopulation called LIC, which is highly resistant to apoptosis following treatment with various chemotherapeutic regimens. Therefore, a major barrier impeding the cure of ATL patients may be the failure to effectively eliminate these LICs. In fact, studies demonstrated that combination therapy using arsenic trioxide and interferon-alpha (IFN) triggers proteasome-mediated Tax proteolysis and apoptosis and cures Tax-driven ATL in mice. This combination therapy of primary donor mice eliminated LIC engraftment and hampered ATL development in untreated secondary recipient mice [20]. Although this treatment showed promising results for long-term remission of ATL patients in the chronic phase of the disease, it did not benefit patients in the acute stage [21].

A study demonstrated that Hoechst-sorted SP cells correspond to CSC/LIC and investigated their role using a Tax-transgenic mouse model that causes T-cell lymphomas with characteristics similar to that of ATL [22]. The authors demonstrated that injection of non-obese diabetic/severe combined immunodeficiency (NOD/SCID) mice with as few as 10^2^ CSC/LIC was sufficient to recapitulate the original lymphoma and reestablish CSC/LIC in recipient NOD/SCID mice, suggesting a role for CSC/LIC in this ATL malignancy. However, it is important to bear in mind the limitations of data derived from transgenic mouse models. Enforced over expression of the Tax oncoprotein in mature T cells is not reflective of the interactions between a complete HTLV-I virus and targeted signaling pathways *in vivo*. Tax expression is limited or absent in many ATL patients [23]. This approach also does not account for the role of the other viral accessory genes, such as p12, p30 and HBZ [24–26], in modulating viral leukemogenesis or playing a role in the CSC/LIC compartment.

In this study, we demonstrated the existence of SP cells in all ATL fresh samples and ATL patient-derived cell lines tested. We used FACS cell sorting to characterize signaling pathways modulated in SP cells and show that the activity of NOTCH1 and Tax, and the expression of STAT3 and β-catenin/Wnt3, are predominantly decreased in ATL SP cells. Consistent with these results, ectopic expression of a constitutive active form of NICD significantly reduced the SP population while, on the other hand, inhibition of NOTCH1 signaling led to enrichment of the SP cells. These results suggest that targeted inhibition of NOTCH1 may reduce tumor burden but may not eliminate CSC/LIC. This is important because numerous studies suggest that leukemia relapse occurs because standard chemotherapy fails to eradicate CSC/LIC [27]. Therefore, elucidating the specific nature and properties of ATL CSC/LIC self-renewal and resistance to apoptosis represents an essential step towards curing ATL.

## Materials and Methods

### Cells and reagents

HTLV-I-transformed cell lines ED, MT1, ATL-T, and ATL-25 were cultured in RPMI-1640 with 10% fetal bovine serum, L-glutamine, 100 U/ml penicillin and streptomycin and maintained in 5% CO_2_ at 37 °C. Vybrant® DyeCycle™ (DCV) was obtained from Invitrogen. Verapamil hydrochloride was purchased from Sigma-Aldrich. ED or MT1 cells were treated with either 10 µM LY294002 (Sigma-Aldrich, St Louis, MO) for 3 days or 1µM gamma-secretase inhibitor (GSI, Calbiochem) for 5 days as indicated in the figure legends.

### Patient samples

ATL cryopreserved samples were obtained after informed consent and institutional IRB approval as described in the previous study [28].

### Side population (SP) analyses

For DCV staining, cells were pelleted and resuspended in pre-warmed DMEM with 10% FBS and 10 mM HEPES at a concentration of 1×10^6^ cells/ml. Before incubation with DCV, cells were pre-incubated for 30 minutes in 200 µM Verapamil at 37 °C. DCV was added at a final staining concentration of 10 µM.

The cells were stained for 60 min at 37 °C while gently vortexing every 15 min. Then the cells were washed 2 times with pre-warmed PBS and resuspended in pre-warmed DMEM with 10% FBS and 10 mM HEPES at a concentration of 1×10^6^ cells/ml. After 1 hour, the cells were either analyzed on a BD™ LSR II cytometer or flow sorted on a BD FACSAria™.

### RNA extraction and Real-Time quantitative RT-PCR

Acute ATL samples were previously published [28]. Total mRNA was isolated from HTLV-I cell lines and cells using TRIzol Reagent (Ambion) according to manufacturer’s instructions. After DNAse I treatment, the RNA was reverse transcribed and the cDNA was used for real-time PCR. Real-time PCR was performed with the following sets of primers:

ABCG2F: CCTGAGATCCTGAGCCTTTGG-3’), ABCG2R: AGGTCATTGGAAGCTGTCGC; Hes1F: CTGTGGGAAAGAAAGTTTGGG; Hes1R: GACCAAGGAGAGAGGTAGAC; HBZF: CGGCCTCAGGGCTGTTTC; HBZR: CGCGGCTTTCCTCTTCTAAGGA; GAPDHF: GAAGGTGAAGGTCGGAGTC; GAPDHR: GAAGATGGTGATGGGATTTC

The relative mRNA levels in each sample were normalized with GAPDH and were calculated using the 2-ΔCt method.

### Production and transduction of recombinant *lentivirus*

*Lentivirus* vector SMPU-18×21-EGFP was kindly provided by Dr. C. Z. Giam [29]. The VSV-G pseudo-typed pSIH-H1-GFP and SMPU-18×21-EGFP viruses were produced and concentrated as previously reported [30]. ATL-25 and MT1 cells were infected in the presence of polybrene and spinoculated at 1200 relative centrifugal force (RCF) at room temperature for 1 hour. The cells were cultured for 2 days, followed by the SP analyses.

### Western blot

MT1 cells were treated with 1µM GSI for 5 days. Whole cell extracts were prepared with radio immune precipitation assay (RIPA) buffer (50 mm Tris-Cl, pH 7.5, 150 mm NaCl, 1% Nonidet P-40, 1% sodium deoxycholate, 0.1% SDS) containing Complete Protease Inhibitor cocktail (Roche Diagnostics). Anti-NOTCH1 (#2421; Cell Signaling) and anti-actin (C-11; Santa Cruz Biotechnology) were used.

## Results

### Characterization of SP cells in ATL fresh samples and patient-derived atl cell lines

**Figure 1:**
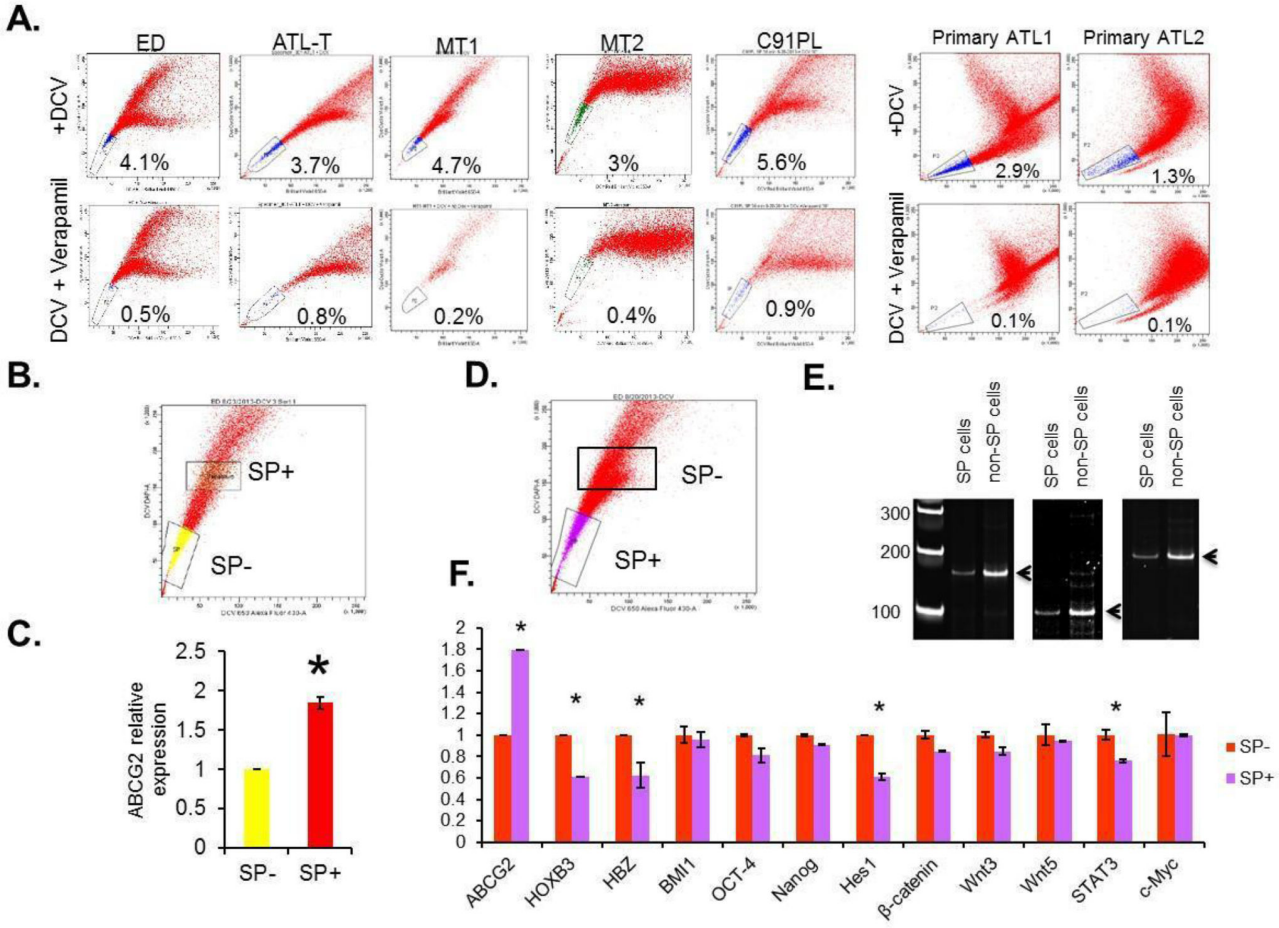
The presence and expression profile of side population in ATL cell lines and primary ATL samples. (A) The representative SP analysis of ATL cell lines and primary ATL samples. (B and C) The gates used to sort both SP+ and SP− cells were plotted as rectangles. The expression of ABCG2 in SP− and SP+ cells were analyzed by RT-PCR. The results were from two independent experiments and normalized to GAPDH expression. Data are mean ± SD. *P<0.05, two-tailed Student’s t-test. (C and D) SP+ and SP− cells were sorted from patient samples. The gates used to sort both SP+ and SP− cells were plotted as rectangles. DNA were extracted and analyzed by PCR. (E) Relative expressions of viral HBZ gene, cellular ABCG2, HOXB3, β-catenin, Hes1, BMI1, OCT-4, Nanog, Wnt3, Wnt5a, STAT3 and c-Myc were tested using RT-PCR. The results were from two independent experiments and normalized to GAPDH expression. Data are mean ± SD. *P<0.05, two-tailed Student’s t-test.

Numerous studies have shown that side population (SP) cells are enriched for cancer stem cells (CSC)/leukemia-initiating cells (LIC), which have both self-renewal and tumor-regenerating potential [3]. The SP phenotype is based on the ability of these cells to proficiently efflux fluorescent dyes such as Hoechst 33342 or DCV through the multidrug ABC transporter, such as ABCG2. This property allows the characterization and isolation of SP cells using fluorescence-activated cell sorting (FACS). To identify and characterize SP cells in ATL, we investigated the SP cells by efflux of DCV dye in several ATL-derived cell lines (ED, ATL-T, MT-1, MT-2 and C91PL) as well as freshly isolated uncultured PBMCs from acute ATL patients. Our results demonstrate the presence of a small percentage of SP cells, from 3% to 5.6%, in all ATL lines and in freshly isolated uncultured ATL primary samples (Figure 1A). Verapamil, an irreversible inhibitor of ABCG2, confirmed loss of SP cells and was used for gating of the cell population in further experiments. We cell-sorted SP cells (SP+) and non-SP cells (SP−) cells and extracted RNA and genomic DNA (Figures 1B–1E), and ABCG2 expression was compared between SP− and SP+ cells in ED. In keeping with earlier reports, there was almost two times the ABCG2 expression in SP+ cells compared with SP− cells (Figure 1C). Notably, PCR-based analyses of T-cell receptor (TCR) gamma gene rearrangement in DNA extracted from SP+ and SP− cell populations indicated that these two populations have the same clonal origin (Figure 1E).

### Signaling pathways involved in the development and maintenance of the malignant ATL cells

Several signaling pathways such as Bmi-1, Notch, Wnt/β-catenin, Sonic hedgehog and NF-kB have been implicated in CSC/LIC self-renewal and survival in leukemia and other solid cancers [31–36]. Previous studies have reported an increased expression of c-Kit and decreased expression of Tax, NOTCH1, and Bmi1 in CSC/LIC isolated from a Tax transgenic mouse model [22]. FACS-sorted SP+ and SP− cells were used to extract RNA for RT-PCR analyses of selected genes previously shown to play a role in the development and maintenance of the malignant ATL cells. Among the targets tested, HOXB3, Hes1, a downstream target of Notch 1, and STAT3 were down regulated in SP+ cells (Figure 1F). Since we have reported somatic mutations of the NOTCH1 and STAT3 signaling pathway in ATL patients [28,34] the effect of these signaling pathways in SP cells should be considered when applying targeted therapy.

Consistent with previous studies, we found no significant change in the expression of FLT3, N-cadherin, Oct-4, and Nanog (Figure 1F and data not shown). In our experiments, however, c-Kit (CD117) expression was not elevated in SP cells, suggesting differences between Tax-derived ATL-like transgenic models and patient-derived ATL cells, highlighting the need for further investigation.

In HTLV-I-transformed ATL cells, the most frequently expressed viral genes are HBZ and Tax. Interestingly, the HBZ mRNA was significantly down regulated in SP+ cells (Figure 1F). These data suggest that loss of HBZ may play a role in the maintenance of SP cells in ATL.

**Figure 2:**
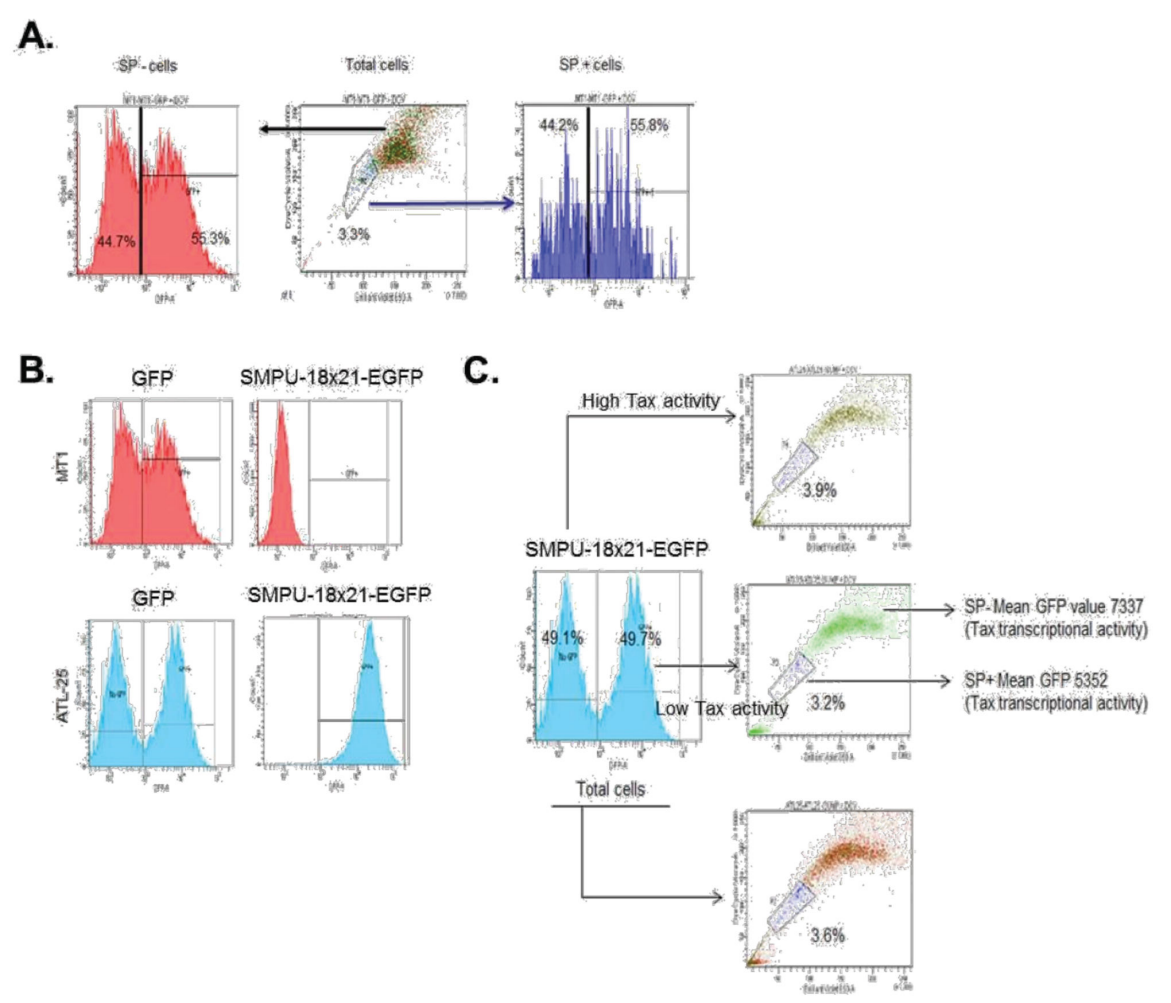
Tax-transcription activity and SP cells. (A) MT1 was first gated for SP+ and SP−, and then the SP+ and SP− cells were gated based on GFP expression level. (B) Both MT1 and ATL-25 were infected with SMPU-18×21-EGFP virus; SMPU-18×21-EGFP can only be detected in Tax+ ATL-25 cells (lower panel), but not Tax− MT1 cells (upper panel). (C) ATL-25 cells are first gated according to Tax-transcription activity and then SP assays were performed to analyze SP cells in each subgroup and total cells.

Next, we sought to analyze viral Tax activity in SP+ and SP− cells. Unlike HBZ mRNA generally expressed in most ATL cells, only about one-fourth of ATL samples have detectable expression of tax viral mRNA [19]. Tax is a potent transcriptional trans-activator for a 21bp repeat motif found in the viral HTLV-I LTR promoter. To detect Tax activity in ATL cells, we used a previously characterized *lentiviral* vector known as SMPU-18×21-EGFP reporter construct [29,30]. We first demonstrated that SP+ and SP− cells are equally susceptible to *lentivirus* infection using concentrated virus particles generated with pSI-H1-GFP. As demonstrated in Figure 2A, approximately 50% of cells in each population were transduced with pSI-H1-GFP. To validate our approach we used Tax− cells (MT1) and Tax+ cells (ATL-25), and measured GFP activity by FACS. Our results suggested that the GFP signal was comparable for both cell lines when using the pSI-H1-GFP particles, which indicated equal transduction efficiency (Figure 2B). However, SMPU-18×21-EGFP signaling was only detected in Tax+ ATL-25 cells, but not Tax− MT1 cells, which demonstrates the specificity of the reporter (Figure 2B). In order to demonstrate the correlation between Tax-transcription activity and SP, we gated the ATL cells according to Tax-transcription activity (SMPU-18×21-EGFP signaling) and then performed SP assay. A lower percentage of SP cells were found in the high Tax-transcription activity population compared with the low Tax-transcription activity population (3.2% vs. 3.9%) (Figure 2C).

### Activated notch signaling depletes SP cells

**Figure 3:**
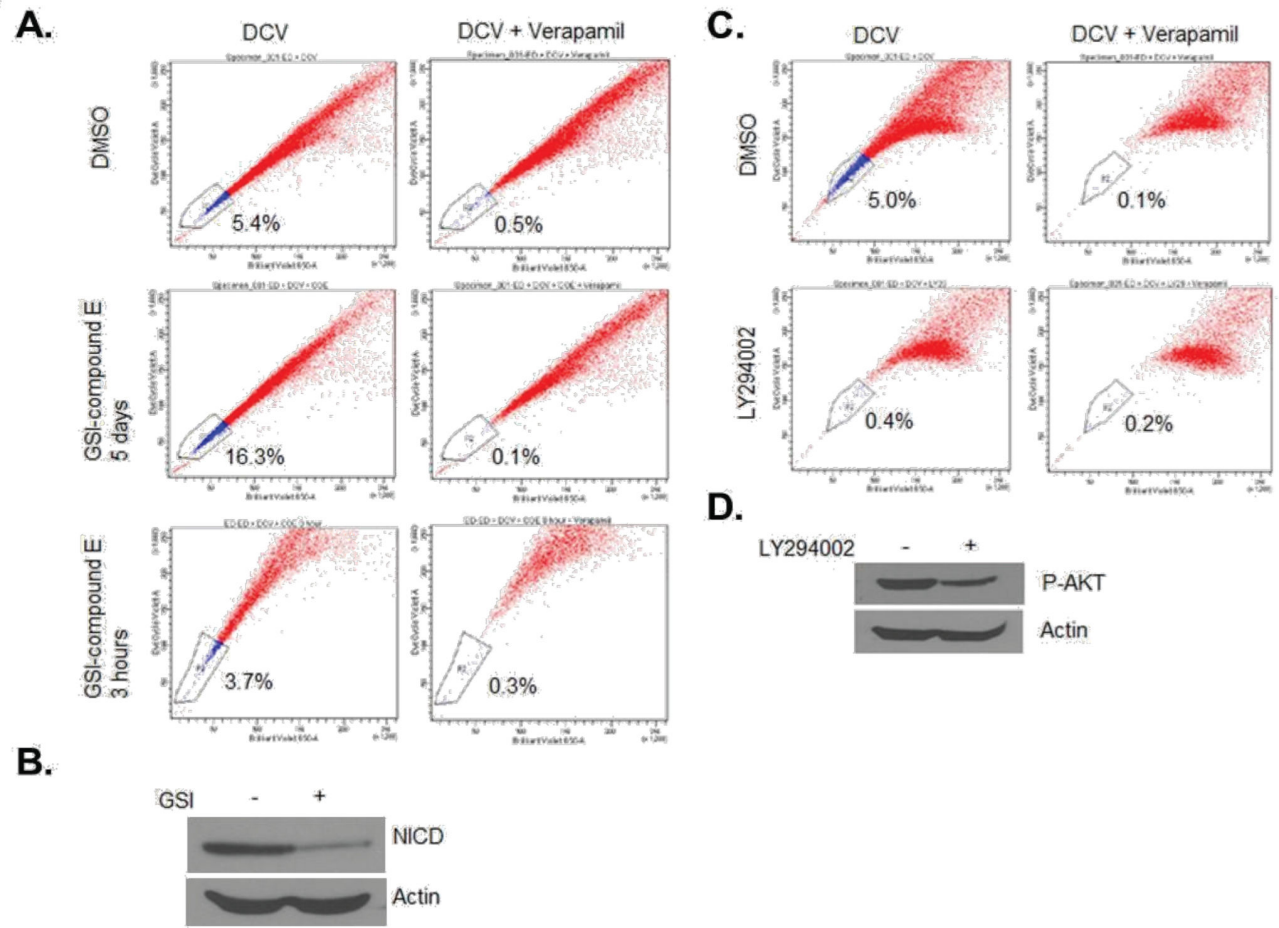
The treatment of LY294002 decreases percentage of SP cells, while the treatment of GSI increases the SP percentage. (A and B) ED cells were treated with 1µM GSI for 5 days or 3 hours, followed by SP analysis (A) and Western blot for cleaved Notch1 (B). Actin served as a loading control. (C and D) ED cells were treated with 10 µM LY294002 for 3 days, followed by SP analysis (C) and Western blot for phospho-AKT (D). Actin served as a loading control.

Previous studies have shown that NOTCH1 signaling plays an important role in CSC/LIC homeostasis [35]. Since our studies revealed Hes-1 as one of most deregulated genes in ATL SP cells and NOTCH1 signaling has been implicated in ATL tumor growth *in vitro* and *in vivo* [28], we next investigated the role of NOTCH1 signaling in SP cells maintenance. Notch signaling, initiated by receptor-ligand interactions, requires subsequent proteolytic cleavage of the receptor, resulting in the intracellular cleaved form of NOTCH1 (hereafter referred to as NICD) which translocates to the nucleus and up-regulates the transcription of Notch-regulated genes (3–5). Treatment with gamma secretase inhibitor (GSI, Calbiochem) prevents cleavage of the receptor and interrupts NOTCH1 signaling. Effectiveness of the treatment was confirmed by decreased NICD expression in western blotting (Val1744 Ab, Figure 3B) following incubation with 1 µM GSI. Treatment of ATL cells with GSI resulted in a significant increase in SP cells from 5.4% to 16.3% (Figure 3A). This increase was not observed after a short incubation of 3 hours with GSI, suggesting that an increase in SP cells is specific to GSI-mediated loss of NICD rather than an effect on ABCG2 pump activity (Figure 3A). In contrast, treatment of cells with LY294002, a PI3K inhibitor, resulted in a drastic loss of SP cells (Figure 3C). The treatment was effective in ATL cells as demonstrated by reduction of pAKT by western blot (Figure 3D). Together these data suggest that the NOTCH1 and PI3K signaling pathways have antagonizing effects on SP cells maintenance.

**Figure 4:**
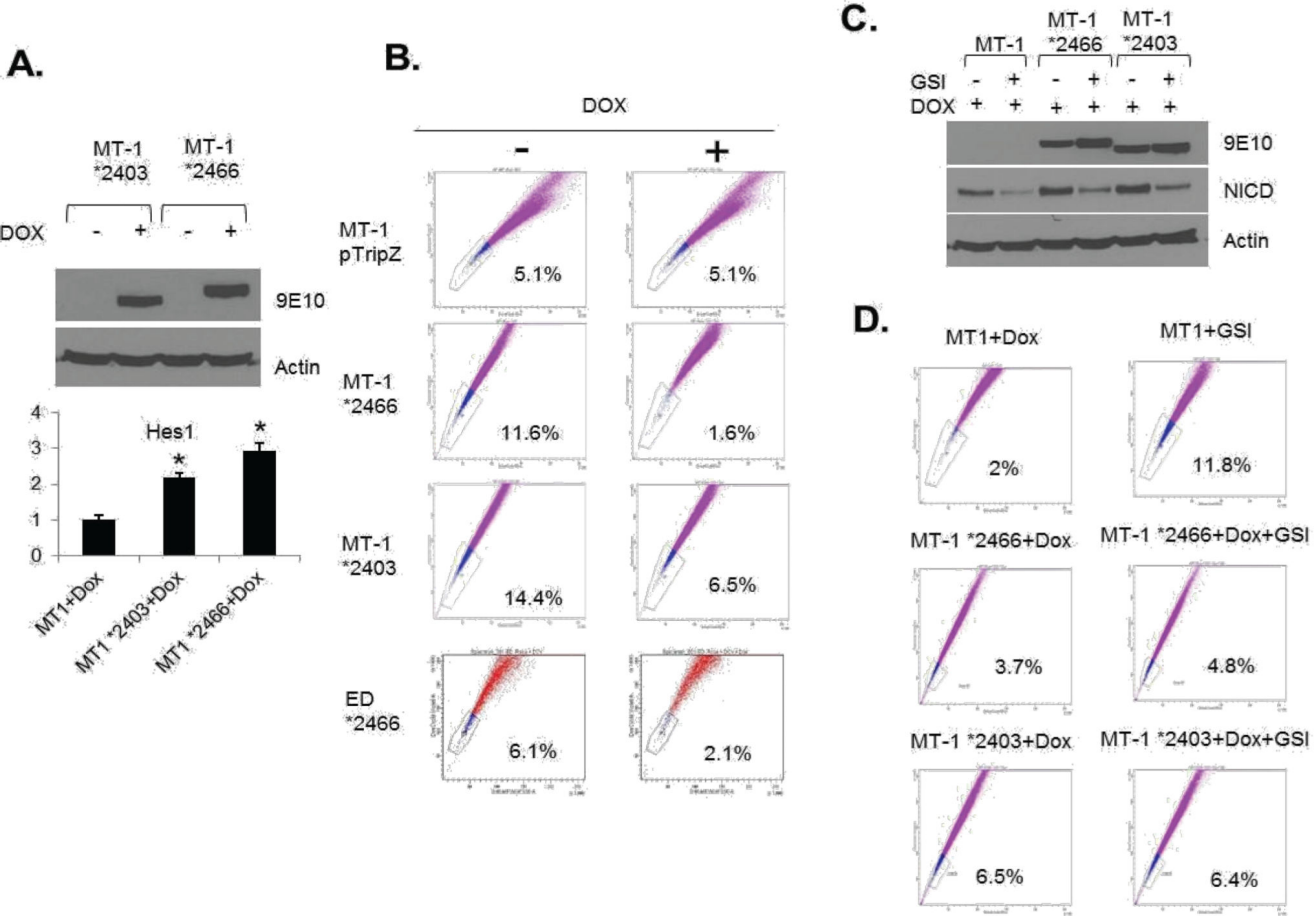
The regulation of SP cells by Notch signaling. (A) MT1 *2403 and *2466 stable cell lines were induced with doxycycline for 48 hours and western blot showed the expression of NICD. Actin acted as a loading control. The expression of Hes1 was analyzed by RT-PCR. The results were from two independent experiments and normalized to GAPDH expression. Data are mean ± SD. *P<0.05, two-tailed Student’s t-test. (B) SP assay was performed after MT1 and ED stable cell lines were induced with doxycycline for 48 hours. (C) Ectopic and endogenous expression of NICD were analyzed by Western blot after indicated treatment. (D) SP assay was performed after MT1 stable cell lines were treated with Dox alone or Dox + GSI.

To eliminate the possibility of GSI off-target effects and further demonstrate the role of NOTCH1 activation in SP cells, we generated Tet-inducible ATL lines carrying a non-degradable constitutive active form of NICD (*2403 and *2466) [28]. Induction of NICD mutant expression (Figure 4A) with Doxycycline was, as expected, associated with increased Hes1 gene expression (Figure 4A). Consistent with results presented in Figure 3, expression of constitutive NOTCH1 in MT-1(MT-1 *2403 and MT-1 *2466) was associated with a significant loss of the SP cells from 11.6% to 1.6% and from 14.4% to 6.5% (Figure 4B). However, the SP cells were not affected in MT1 control cells (MT-1 pTripZ) (Figure 4B). These results were further confirmed in a different ATL patient-derived cell line (ED) using the *2466 NICD mutant (ED *2466) (Figure 4B). Finally, if the GSI effect described above occurs through NOTCH1 signaling, one would expect that expression of NICD mutants *2403 and *2466 would prevent an increase of the SP cells following GSI treatment. Western blot analyses confirmed that *2403 and *2466 expression is not affected by GSI, while endogenous levels of NICD are reduced after treatment (Figure 4C). FACS analyses further demonstrate that the SP cells significantly increases only in MT1 control ATL cells (2% to 11.8%) (Figure 4D) but not in MT1 cells carrying constitutive active NICD *2466 and *2403 mutants (3.7% to 4.8% and 6.5% to 6.4%, respectively) (Figure 4D). Altogether our studies suggest that activation of NOTCH1 is critical for SP cells maintenance.

## Discussion

HTLV-I-associated ATL has limited therapeutic options, a very poor prognosis and a dismal survival rate. The 4-year survival rate for acute-, lymphoma-, chronic- and smoldering-type ATL is 5.0, 5.7, 26.9 and 62.8%, respectively [17]. The poor prognosis of ATL patients is associated with the resistance of tumor cells to the conventional combination of high-dose chemotherapy and radiotherapy, in addition to ATL being associated with a high rate of disease relapse. The failure of first line therapies to completely eliminate cancer cells likely contributes to acquisition of chemo resistance [37]. Hence, a better understanding of cell population and the genetic and epigenetic events providing chemo resistance is critical for the design of novel therapeutic strategies to successfully treat cancer. In recent years, the role of CSC/LIC in cancer resurgence and resistance to treatment has been the focus of many investigations. High levels of ABC-transporter-mediated efflux, such as ACBG2, facilitate but do not solely explain the acquisition of mechanisms of drug resistance. Defects in DNA repair pathways, control of apoptosis cell death and genetic mutations observed in ATL cells [38] may be contributing factors leading to CSC/LIC escape from chemotherapy. CSC/LIC has been poorly characterized in HTLV-I-associated ATL.

In this study, we demonstrate that HTLV-I-transformed ATL cells freshly isolated or cell lines derived from patient samples contain a small variable population of SP cells. As mentioned above, SP analysis has been used to identify CSC/LIC in a wide variety of leukemia and solid tumors. Our studies demonstrate that ATL SP cells display a lower expression of Hes1, STAT3, β-catenin and Wnt3. This is of interest because we have previously identified a high rate of somatic mutations in NOTCH1 and STAT3 in acute ATL patients [28,34]. These mutations could trigger rapid proliferation and expansion of leukemia cells. Whether mutations occur in the CSC/LIC or leukemic cellular compartment remains to be determined. In contrast, we have previously shown that ATL patient-derived leukemia cells activate the non-canonical Wnt pathway and over express Wnt5 but do not present activation of β-catenin or Wnt3 [31]. In this study, we also investigated the functional expression of the two viral genes most frequently expressed in ATL cells isolated from patients, Tax and HBZ. Our investigations revealed a reduced expression of viral HBZ and lower Tax activity in SP+ cells. These data are consistent with the fact that CSC/LIC is slowly dividing cells and viral proteins have an opposite effect. Tax is a potent transcriptional activator of cellular genes involved in cell proliferation and it favors genome instability by targeting DNA repair pathways [38–40]. Similar to Tax, HBZ has been shown to stimulate the growth of ATL cells and activate the non-canonical NF-kB pathway. Our investigations suggest that the PI3K and NOTCH1 signaling pathways have opposite functions in SP cells homeostasis. While constitutive activation of NOTCH1 signaling depletes the pool of SP cells in ATL-derived cell lines, PI3K signaling seems to increase the pool of SP cells. Additional experiments will be needed to further characterize the role of these signaling pathways in CSC/LIC and identify effective therapeutic targets. While inhibition of the NOTCH1 signaling pathway may be effective in eliminating ATL leukemia cells, this strategy may increase the SP cells and lead to disease relapse, suggesting that combination therapies targeting both cellular compartments may be more effective in curing ATL.
